# 

**Published:** 1904-06

**Authors:** 


**This Journal and Atlanta Semi-weekly Journal one year for $1.25 cash in advance. No checks. Strictly to new subscribers.**

THE Fifty-fifth Annual Meeting of the American Medical Association will be held at Atlantic City, June 7, 8, 9, 10.

Nothing has been spared to make the meeting attractive, and the popularity of Atlantic City as a summer resort will have its own attractive force. The hotels are numerous and the prices normal. The railroads offer reduced rates, and it is hoped that many from Georgia will attend the meeting and identify themselves with the spirit and aim of this increasingly powerful association.

**This Journal and Atlanta Semi-weekly Journal one year for $1.25 cash in advance. No checks. Strictly to new subscribers.**

**NOTICE.—This JOURNAL and THE INTERNATIONAL JOURNAL OF SURGERY one year for $1.00 cash, to new subscribers. No out-of-town Checks accepted unless 10c. is added for cost of cashing.**

**This Journal and Atlanta Semi-weekly Journal one year for $1.25, cash in advance. No checks. Strictly to new subscribers.**

